# Quantitative proteomic analysis of gastric cancer tissue reveals novel proteins in platelet-derived growth factor B signaling pathway

**DOI:** 10.18632/oncotarget.15908

**Published:** 2017-03-06

**Authors:** Fang Liu, Yuan Zhang, Tingting Men, Xingyue Jiang, Chunhua Yang, He Li, Xiaodan Wei, Dong Yan, Gangming Feng, Jianke Yang, Jonas Bergquist, Bin Wang, Wenguo Jiang, Jia Mi, Geng Tian

**Affiliations:** ^1^ Medicine and Pharmacy Research Center, Binzhou Medical University, Yantai, Shandong Province, 264003 China; ^2^ Department of Radiology, Affiliated Hospital of Binzhou Medical University, Binzhou, Shandong Province, 256603 China; ^3^ Department of Chemistry – BMC, Uppsala University, Uppsala, 75124, Sweden; ^4^ Department of Gastric and Intestine, Yantai Affiliated Hospital of Binzhou Medical University, Yantai, Shandong Province, 264003 China; ^5^ Yantai Institute, China Agriculture University, Yantai, Shandong Province, 264670 China

**Keywords:** proteomic, PDGF-B, gastric cancer, PRDX5, pathway

## Abstract

Gastric cancer is one of the most common cancers in Asian countries. Searching for reliable biomarkers involving the development of gastric cancer is important for clinical practice. Quantitative proteomics has become an important method contributed to the discovery of novel diagnostic or therapeutic targets for the management of cancer. Here, we identified differently expressed proteins in gastric cancer and normal gastric tissues by using the high resolution mass spectrometer. Among the total of 2280 identified proteins, 87 were differentially expressed between gastric cancer and normal gastric tissues. Notably, several significant proteins are in the PDGF-B signaling pathway, including peroxiredoxin5 (PRDX5), S100A6, calreticulin (CALR) and cathepsin D (CTSD), which were validated by western blot. Furthermore, upstream regulators including PDGF-B, PDGFR-β, Akt, eIF4E and p70s6K were found significantly increased in the gastric cancer tissues. In addition, silencing of PRDX5 and PDGF-B suppressed the proliferation of gastric cancer cells *in vitro*. The administration of exogenous PDGF-BB recovered the reduced expression of PDGF-B signaling pathway in PDGF-B knockdown cells. Taken together, our findings suggested that PDGF-B signaling pathway plays an important role in the regulation of gastric cancer proliferation and the inhibition of this pathway may be a potential approach for treatment of gastric cancer.

## INTRODUCTION

Gastric cancer (GC) is one of the most common cancers in Asian countries and is the fourth most commonly occurring cancer worldwide (9% of all cancers) [1–3]. If GC is detected and treated early, the 5-year survival rate is better than 90% [4]. In contrast, the 5-year survival rate of patients with advanced-stage gastric cancer is less than 10% [5].

It is thus important to have biomarkers for the early diagnosis and follow-up of GC. Unfortunately, diagnostic biomarkers in GC, such as the carcinoembryonic antigen (CEA), carbohydrate antigen 19-9 (CA 19-9), and carbohydrate antigen 72-4 (CA72-4), are neither specific nor sensitive enough [6–9]. The evolution of proteomic technologies has enabled not only the screening of a large number of samples, but also the identification of pathologically significant proteins, including phosphoproteins, and the quantitation of differences in protein expression under different conditions [10, 11]. In the past decade, numerous groups have attempted to profile the expression changes in GC compared with normal gastric tissues using proteomic approaches to search for diagnostic and prognostic biomarkers [12, 13]. However, a more deeper analysis is still in urgent need.

Proteomic technologies have been widely used to fractionate complex samples such as saliva, blood, tissue, cells, and *Helicobacter pylori*-infected specimens and identify differentially expressed proteins [14–19]. In particular, paired GC and normal gastric tissues obtained after surgical resection are usually analyzed by labeling or label-free Mass Spectrometry (MS) [12, 20, 21]. A number of potential prognostic markers, such as filamin C, human epidermal growth factor receptor 2 (HER2) and human neutrophil peptides 1-3 (HNPs 1-3) have been identified on the basis of proteomic analyses [22–25]. Therefore, proteomic analyses are powerful tools for the identification of key molecules involved in the development of GC and prognostic factors for GC patients.

The aim of this study is to discover reliable protein biomarkers from GC and adjacent normal tissues based on label free liquid chromatography (LC)-MS proteomic analysis, and investigate novel regulation network in GC. We evaluated GC and normal gastric tissues from 6 patients and focused on proteins associated with the PDGF pathway that were differentially expressed between GC and normal gastric tissues in all 6 patients. PDGF-B and PDGFR-β have been demonstrated earlier to be expressed in many kinds of cancers. These proteins are emerging as key regulators of mesenchymal cells in the tumor microenvironment and many common malignancies. High stromal PDGFR-β expression or activation is associated with a poor prognosis in breast and prostate cancers [26, 27]. Data-mining using publicly available gene microarray datasets also showed that changes in the PDGF-B pathway were significantly associated with human primary and metastasis cancer.

## RESULTS

### Protein identification and quantification by label-free LC-MS/MS

**Figure 1:**
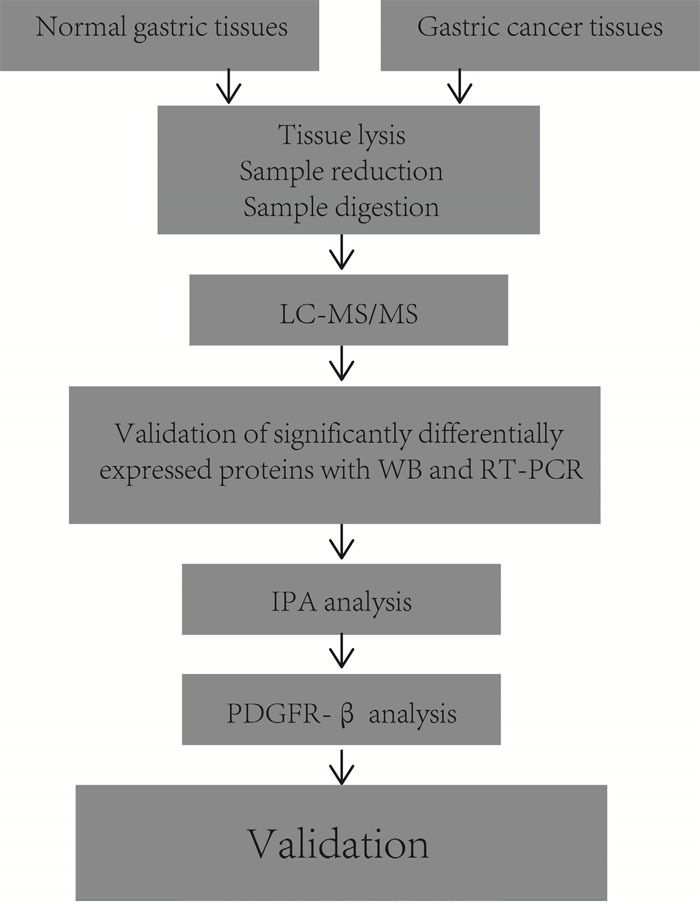
Experimental design for proteomic analysis in human gastric cancer tissues by label-free LC-MS

The experimental design is briefly shown in Figure 1. To explore global differences in protein expression between GC and normal tissues, proteins were extracted from surgically resected fresh GC and adjacent tissues of 6 patients after in-solution tryptic digestion. Each of the 6 pairs of samples was analyzed in triplicate by LC-MS/MS. A total of 2280 proteins were identified and quantified by MaxQuant 1.5.0.1. In total, 87 proteins were identified as being significantly differentially expressed between the GC and normal tissues in all 6 patients (*p*<0.05). Of these proteins, 65 were upregulated and 22 were downregulated. Proteins upregulated in GC tissue included PRDX5, ATP5A1, calreticulin (CALR), and cathepsin D (CTSD). Proteins downregulated in GC tissue included S100A6, vinculin, and annexin A1. Table 1 a lists 14 proteins that were detected in all 6 GC tissues but never identified in adjacent tissues. Table 1 b displays 73 proteins that were differentially expressed between GC and normal gastric tissues.

Table 1

**Table:** a. List of 14 proteins that expressed only in gastric cancer tissues

ID	Protein names
P42677	ribosomal protein S27
Q02790	FK506 binding protein
Q9UIJ7	adenylate kinase 3
P35914	3-hydroxymethyl-3-methylglutaryl-CoA lyase
P56381	ATP synthase, H+ transporting, mitochondrial F1 complex, epsilon subunit
Q9P2R7	succinate-CoA ligase, ADP-forming, beta subunit
P55809	3-oxoacid CoA transferase 1
P18859	ATP synthase, H+ transporting, mitochondrial Fo complex, subunit F6
Q9P0J0	NADH dehydrogenase (ubiquinone) 1 alpha subcomplex, 13
Q9Y6M9	NADH dehydrogenase (ubiquinone) 1 beta subcomplex
P08263	glutathione S-transferase alpha 1
P51164	ATPase, H+/K+ exchanging, beta polypeptide
Q16822	phosphoenolpyruvate carboxykinase 2 (mitochondrial)
Q96NY7	chloride intracellular channel 6

**Table:** b. List of 73 proteins that different expressed in gastric cancer tissues and normal gastric tissues

IDs	Protein names	pvalue	Ratio
P46777	60S ribosomal protein L5	2.23E-06	2.8549
P07339	Cathepsin D;Cathepsin D light chain;Cathepsin D heavy chain	1.36E-04	1.6304
P23246	Splicing factor, proline- and glutamine-rich	1.50E-04	1.2438
P23284	Peptidyl-prolyl cis-trans isomerase B	1.56E-04	1.8987
P06703	Protein S100-A6	1.71E-04	0.3656
Q9UL46	Proteasome activator complex subunit 2	1.90E-04	1.7142
P11021	78 kDa glucose-regulated protein	2.71E-04	1.8068
P52597	Heterogeneous nuclear ribonucleoprotein F;Heterogeneous nuclear ribonucleoprotein F, N-terminally processed	3.25E-04	1.6957
P40925	Malate dehydrogenase, cytoplasmic;Malate dehydrogenase	3.72E-04	4.5484
P62913	60S ribosomal protein L11	3.75E-04	2.0507
Q06323	Proteasome activator complex subunit 1	4.50E-04	1.8409
P62888	60S ribosomal protein L30	6.91E-04	2.2881
P0C0L4	Complement C4-A;Complement C4 beta chain;Complement C4-A alpha chain;C4a anaphylatoxin;C4b-A;C4d-A;Complement C4 gamma chain	7.81E-04	0.5634
P14625	Endoplasmin	8.53E-04	2.0368
P25705	ATP synthase subunit alpha, mitochondrial;ATP synthase subunit alpha	9.11E-04	6.0099
Q9P2E9	Ribosome-binding protein 1	9.15E-04	1.7208
P27797	Calreticulin	1.36E-03	1.6196
P61604	10 kDa heat shock protein, mitochondrial	1.70E-03	2.5209
P02790	Hemopexin	1.98E-03	0.5928
Q8NBS9	Thioredoxin domain-containing protein 5	2.63E-03	2.7525
P31930	Cytochrome b-c1 complex subunit 1, mitochondrial	2.74E-03	4.1408
P06576	ATP synthase subunit beta, mitochondrial;ATP synthase subunit beta	2.99E-03	3.4749
P30048	Thioredoxin-dependent peroxide reductase, mitochondrial	4.10E-03	1.7795
Q15084	Protein disulfide-isomerase A6	4.44E-03	2.0287
P60174	Triosephosphate isomerase	5.47E-03	1.278
P01860	Ig gamma-3 chain C region	5.56E-03	0.602
Q15233	Non-POU domain-containing octamer-binding protein	5.59E-03	1.2969
P08865	40S ribosomal protein SA	7.13E-03	2.0812
P02774	Vitamin D-binding protein	7.37E-03	0.5531
P00450	Ceruloplasmin	7.87E-03	0.6645
P30044	Peroxiredoxin-5, mitochondrial	8.35E-03	3.0094
P10606	Cytochrome c oxidase subunit 5B, mitochondrial	8.74E-03	3.4106
P07195	L-lactate dehydrogenase B chain;L-lactate dehydrogenase	9.71E-03	2.232
P15311	Ezrin	1.04E-02	2.4283
Q14624	Inter-alpha-trypsin inhibitor heavy chain H4;70 kDa inter-alpha-trypsin inhibitor heavy chain H4;35 kDa inter-alpha-trypsin inhibitor heavy chain H4	1.13E-02	0.5477
P49748	Very long-chain specific acyl-CoA dehydrogenase, mitochondrial	1.20E-02	3.6355
P48735	Isocitrate dehydrogenase [NADP], mitochondrial;Isocitrate dehydrogenase [NADP]	1.25E-02	12.453
P01766	Ig heavy chain V-III region BRO;Ig heavy chain V-III region WEA;Ig heavy chain V-III region TEI	1.39E-02	0.7126
P14854	Cytochrome c oxidase subunit 6B1	1.42E-02	3.1058
Q16555	Dihydropyrimidinase-related protein 2	1.49E-02	0.7761
P12814	Alpha-actinin-1	1.53E-02	0.4607
P99999	Cytochrome c	1.74E-02	3.0151
P13639	Elongation factor 2	1.95E-02	1.8645
P02763	Alpha-1-acid glycoprotein 1	1.97E-02	0.5033
P30086	Phosphatidylethanolamine-binding protein 1;Hippocampal cholinergic neurostimulating peptide	1.98E-02	2.64
P62805	Histone H4	2.05E-02	1.8766
P00738	Haptoglobin;Haptoglobin alpha chain;Haptoglobin beta chain	2.28E-02	0.6918
P17858	6-phosphofructokinase, liver type	2.59E-02	1.4459
P09525	Annexin A4;Annexin	2.69E-02	1.4307
P10599	Thioredoxin	2.70E-02	1.5319
Q99623	Prohibitin-2	2.77E-02	1.873
P02679	Fibrinogen gamma chain	2.99E-02	0.1522
P18206	Vinculin	3.00E-02	0.6009
P38646	Stress-70 protein, mitochondrial	3.08E-02	1.9969
P02751	Fibronectin;Anastellin;Ugl-Y1;Ugl-Y2;Ugl-Y3	3.16E-02	0.0773
Q15365	Poly(rC)-binding protein 1	3.24E-02	1.5949
P10809	60 kDa heat shock protein, mitochondrial	3.32E-02	1.7442
P01859	Ig gamma-2 chain C region	3.33E-02	0.7329
O43707	Alpha-actinin-4	3.48E-02	0.8575
P45880	Voltage-dependent anion-selective channel protein 2	3.58E-02	1.9714
Q00325	Phosphate carrier protein, mitochondrial	3.58E-02	2.9037
P23528	Cofilin-1	3.73E-02	2.5925
P30101	Protein disulfide-isomerase A3	3.79E-02	1.6033
P68431	Histone H3.1;Histone H3.1t	3.84E-02	1.7335
P02675	Fibrinogen beta chain;Fibrinopeptide B;Fibrinogen beta chain	3.89E-02	0.1133
P40121	Macrophage-capping protein	3.95E-02	0.7774
P50395	Rab GDP dissociation inhibitor beta	4.03E-02	0.8337
P49755	Transmembrane emp24 domain-containing protein 10	4.08E-02	1.3228
P61586	Transforming protein RhoA	4.21E-02	1.2987
P61158	Actin-related protein 3	4.41E-02	1.2005
P21796	Voltage-dependent anion-selective channel protein 1	4.55E-02	2.029
P04083	Annexin A1	4.57E-02	0.3813
670	Vimentin	4.84E-02	0.6984

Total proteins identified from six cases in gastric cancer or normal tissues respectively. A total of 2280 unique proteins were identified. 88 proteins were differential expression proteins with 14 proteins expressed only in gastric cancer tissues.

### Cellular and molecular functional characteristics of the proteins

**Figure 2:**
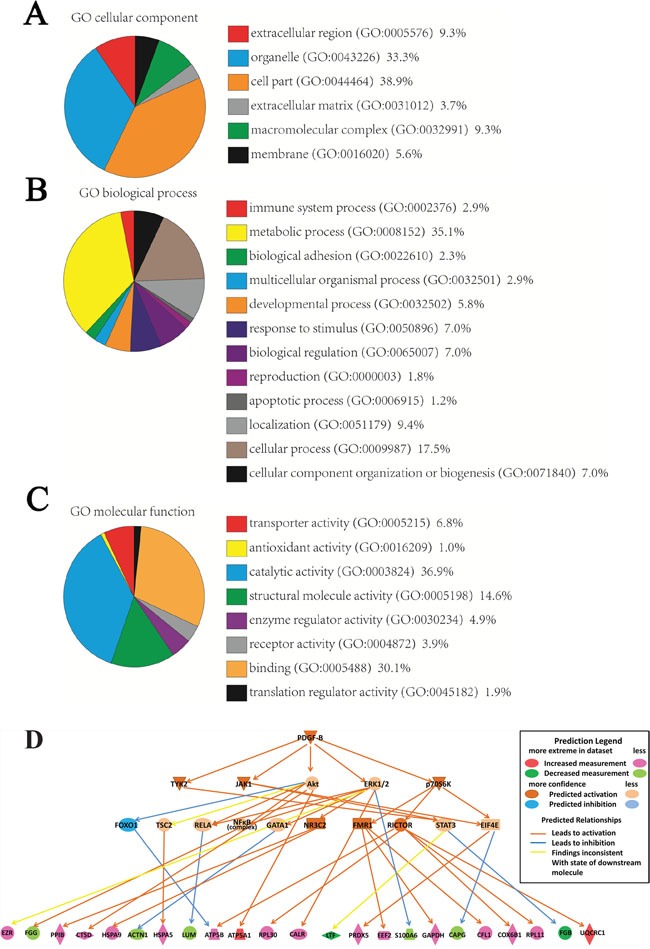
Classification of proteins identified through proteomics into their (A) cellular components, (B) molecular biological processes, and (C) molecular functions This was done via the PANTHER (Protein Analysis through Evolutionary Relationships) Classification System (pantherdb.org). A predictive novel PDGF-B regulation network in gastric cancer progression **(D)**.

To identify the functions of these proteins, the 87 differentially expressed proteins were uploaded into PANTHER (pantherdb.org) and grouped on the basis of their reported cellular components, biological processes and molecular functions. The identities of the 87 proteins were shown in Figure 2A-2C. These proteins were found to cluster into 6 cellular components, 12 biological processes and 8 molecular functions.

### Network analysis of differentially expressed proteins by IPA

We used the IPA software to map the canonical biological pathways represented by the 87 differentially expressed proteins. 30 pathways were significantly overlapped with the 87 proteins, in which several of these pathways are known to be involved in GC, for example, mitochondrial dysfunction, unfolded protein response, and oxidative phosphorylation (Supplementary Figure 1). From the network analysis, PDGF family and PDGF-BB are the two major missing nodes in the network (Supplementary Figure 2). The causal regulation analysis indicated that PDGF-B signaling pathway was significantly involved the cancer progress (P<0.001, Zscore =2.7) and predicted a novel PDGF-B regulation network in GC including 23 differentially expressed proteins (Figure 2D).

### Validation of differentially expressed proteins

**Figure 3:**
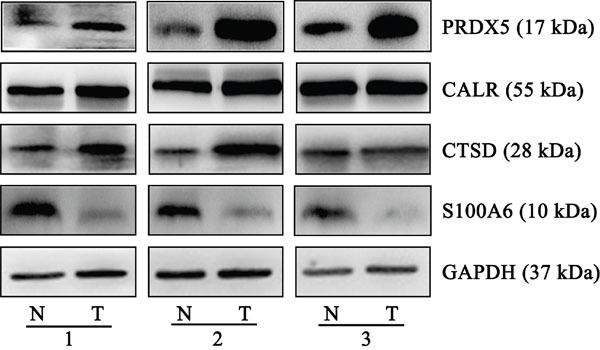
Representative western blots of PRDX5, CALR, CTSD and S100A6 in gastric cancer and normal tissues Compared with normal tissues, gastric cancer tissues from three individual pairs presented up-regulation of PRDX5, CALR, and CTSD, and a marked down-regulation of S100A6. The experiments were repeated at least three times, N represents normal tissue and T represents tumor tissue.

From the *in silico* prediction of PDGF regulation, we selected 4 differentially expressed proteins that were associated with the PDGF-B signaling pathway for further validation. These proteins were PRDX5, CALR, CTSD, and S100A6. Compared with normal tissues, GC tissues showed an obvious upregulation of PRDX5, CALR, and CTSD, and a marked downregulation of S100A6. The trends in the expression of these proteins were similar to those previously determined using proteomics approach. Figure 3 shows representative results from the western blot analyses of PRDX5, CALR, CTSD, and S100A6 expression in GC and normal tissues. Supplementary Figure 3 shows the quantification of the band intensity.

### Confirmation of the differential expression upstream regulator associated with the PDGF-B signaling pathway between GC and normal gastric tissues

**Figure 4:**
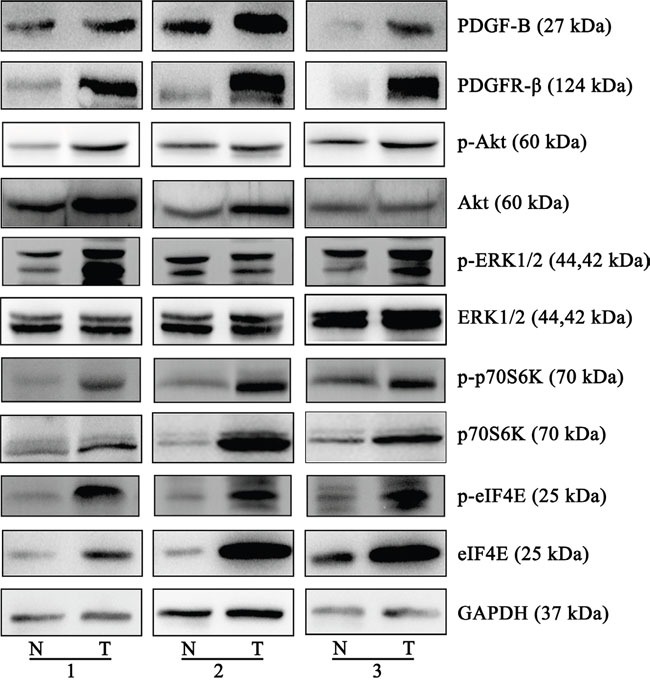
Western blots of the proteins of PDGF-B signaling pathway The different expressions from three individual patients are presented for the selected proteins. PDGF-B, PDGFR-β, eIF4E and p70s6K were increased in the gastric cancer tissues, compared to normal gastric tissues, Akt expression is increased in two groups, ERK1/2 expression was not significantly altered. The phosphorylation and activation status of Akt, ERK, p70S6K and eIF4E were observed significantly increased. All the experiments were repeated at least three times, N represents normal tissue and T represents tumor tissue.

Six upstream regulators of the PDGF-B signaling pathway were predicted to be involved in GC by the proteomic profiling analysis. Of these, the 5 proteins PDGF-B, PDGFR-β, Akt, eIF4E, and p70S6K were increased in the GC tissues compared with normal gastric tissues, whereas the expression of ERK1/2 showed no significant change (Figure 4). Moreover, the phosphorylation and activation status of Akt, ERK, p70S6K and eIF4E were investigated and found that Akt and ERK phosphorylation were more significant active. Supplementary Figure 4 shows the quantification of the band intensity (Supplementary Figure 4).

### PRDX5 plays a role in GC cell proliferation

**Figure 5:**
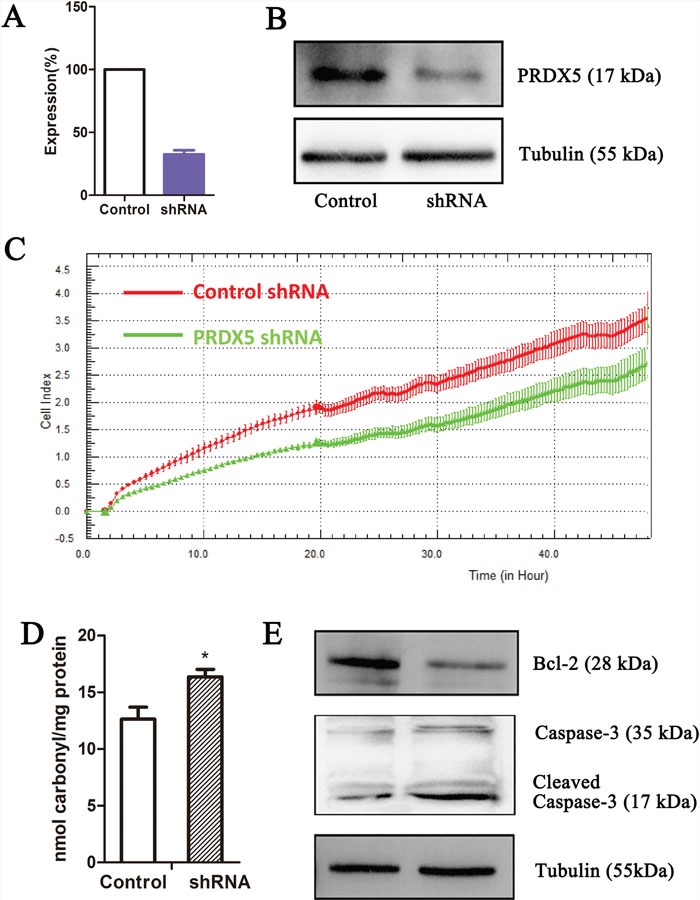
PRDX5 knockdown results in growth inhibition of gastric cancer cell line PRDX5 knockdown as measured by quantitative reverse-transcription PCR **(A)** and western blot **(B)**. Label-free real-time quantitative monitoring **(C)** of cell index over 48 h for SGC7901 cells with shRNAs against PRDX5 (Green) or negative controls (Red). The levels of protein carbonyl contents were measured in PRDX5 knockdown cells **(D)**. The Bcl-2 and Caspase-3 were examined through Western blots **(E)**.

Since we observed that the upregulation of PRDX5 in GC is a frequent event and closely associated with GC development, we postulated that knockdown of *PRDX5* in GC cells would inhibit GC cell proliferation. Thus, we used shRNA technology to inhibit *PRDX5* expression in GC cell line. SGC7901 cells were transfected with *PRDX5*-specific shRNA plasmid. According to real-Time PCR and western blot analysis, efficient silencing of *PRDX5* expression was demonstrated (Figure 5A–5B). As shown in Figure 5C, the downregulation of *PRDX5* markedly inhibited the proliferation of SGC7901 cells, compared with that of the negative controls. PRDXs play an important role in cellular protection against oxidative stress which can lead to cell death. After knockdown of PRDX5, protein carbonyl contents, as an indicator of protein oxidation, were measured. The levels of protein carbonyl contents were increased in PRDX5 knockdown cells (Figure 5D). Furthermore, the Bcl-2 and Caspase-3 were examined. The Bcl-2 expression decreased and the Cleaved Caspase-3 increased (Figure 5E).

### PDGF-B affects GC cell proliferation

**Figure 6:**
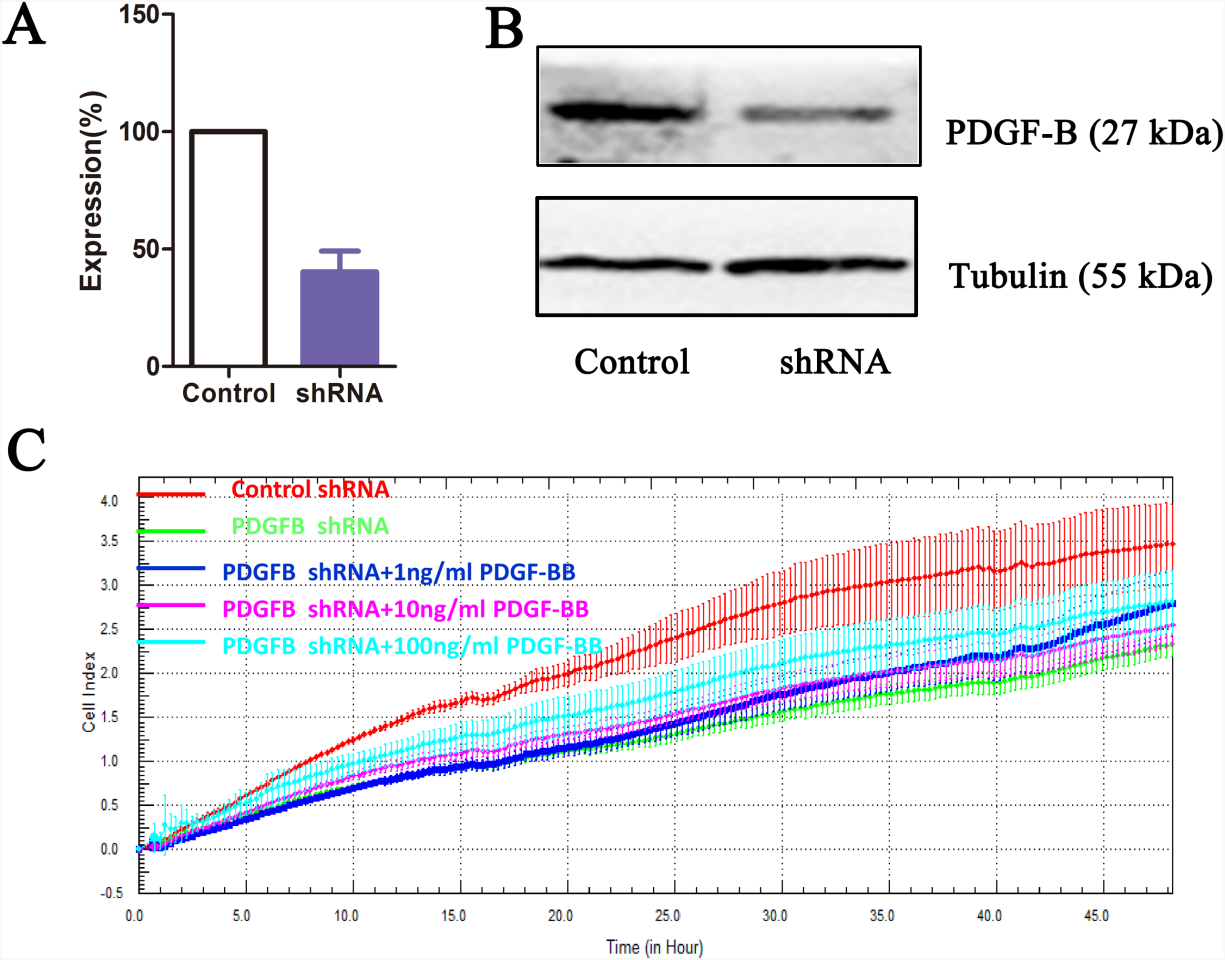
PDGF-B knockdown results in growth inhibition of gastric cancer cell line PDGF-B knockdown as measured by quantitative reverse-transcription PCR **(A)** and western blot **(B)**. Label-free real-time quantitative monitoring **(C)** of cell index over 48 h for SGC7901 cells with shRNAs against PDGF-B (Green) or negative controls (Red).

**Figure 7:**
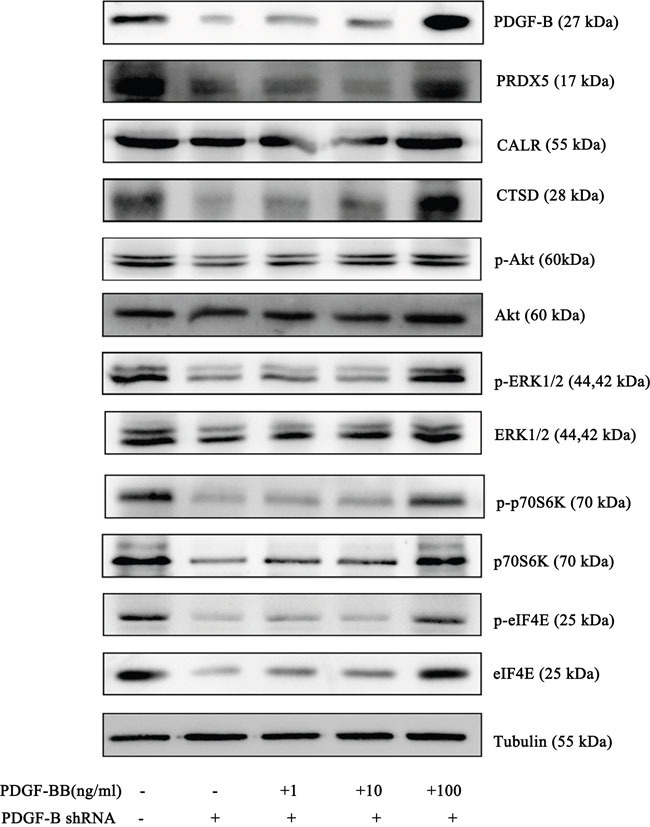
Western Blot results of PRDX5, CTSD, eIF4E, p70S6K and Akt in negative control cells, PDGF-B Knockdown cells and knockdown cells with exogenous PDGF-BB Western blot experiments were replicated three times and the representative results are presented

**Figure 8:**
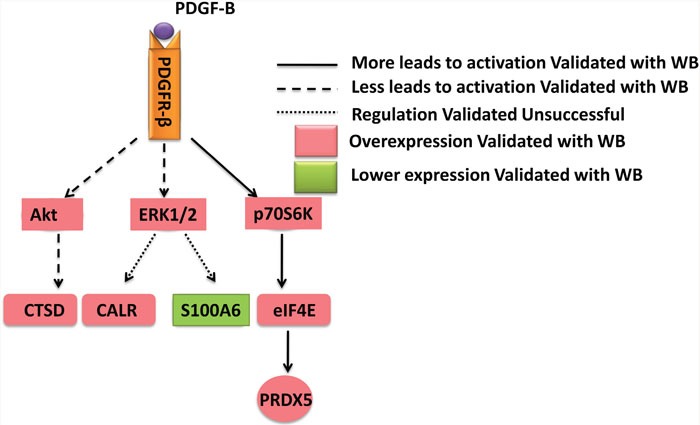
A schematic diagram of PDGF-B signaling network in gastric cancer PRDX5, CALR, CTSD, and S100A6 were identified in the proteomics analysis and validated with western bot. It was confirmed PRDX5 and CTSD were regulated by the PDGF-B signaling pathway.

To determine whether PDGF-B activation influences GC cell proliferation, we used shRNA technology to inhibit *PDGFB* expression in SGC7901 cells. According to real-time PCR and western blot analyses, we demonstrated the knockdown of *PDGFB* mRNA and protein in SGC7901 cells and showed significant effects on cellular proliferation (Figure 6A–6C). To further confirm the regulation effect, cells with knockdown of *PDGFB* were grown in medium containing PDGF-BB (PDGF-B homodimer). PDGF-BB rescued the cellular proliferation in a dose-dependent manner (Figure 6C). Western blot results indicated that the protein expression of PRDX5, CTSD, Akt, p-Akt, p70S6K, p-p70S6K, eIF4E and p-eIF4E was reduced in PDGF-B knockdown cells, and this reduction was recovered by adding exogenous PDGF-BB (Figure 7). Based on the results of the validation processes performed in this study, we propose a PDGF-B signaling network in GC (Figure 8).

## DISCUSSION

In a previous study, Ran et al. identified 134 proteins to be differentially expressed between the GC and non-cancerous gastric tissue samples through the isobaric tags for relative and absolute quantitation (iTRAQ^®^) method [20]. Dai et al. identified 146 proteins that were differentially expressed between GC tumor and adjacent normal tissues by label-free MS approach [12]. In our study, we comparatively analyzed the proteomic profiles of GC and normal gastric tissues and combined with advanced bioinformatics approach to investigate the potential regulation network. We used a QExactive Plus Orbitrap™ mass spectrometer equipped with a nano-electrospray ion source to identify differently expressed proteins. A total of 2280 unique proteins were identified. Among these, 87 were potential differentially expressed between GC and normal gastric tissues. In further pathway analysis, the 4 differentially expressed proteins PRDX5, CALR, CTSD, and S100A6, were predicted to be significantly associated with PDGF-B signaling pathway.

Peroxiredoxins (PRDXs) are a new type of antioxidant protein that catalyzes the reduction of some reactive oxygen species. PRDXs not only play an important role in detoxification, but also increase cell survival and proliferation under conditions of oxidative stress. The expression of PRDXs is closely associated with human cancers such as thyroid cancer, breast cancer, and lung cancer [28]. PRDX5 is an atypical, cytosolic type PRDX, which possesses more effective antioxidant activity against ROS than other PRDXs. It may confer protection against mitochondrial or nuclear DNA damage [29]. Moreover, cells with a reduced expression of PRDX5 were shown to be more prone to oxidative damage and apoptosis. However, there have been no prior published data suggesting that PRDX5 are related with PDGF B signaling. Our results have shown the existence of this relationship for the first time. In a previous study, the overexpressing human PRDX5 inhibited hydrogen peroxide accumulation induced by PDGF [30]. In this study, our functional investigation of PRDX5 using shRNA revealed that knockdown of PRDX5 in GC cells can inhibit GC cell proliferation. These findings suggest that PRDX5 may serve as a candidate diagnostic marker and therapeutic target in GC.

The upstream regulators of PRDX5 were analyzed using IPA. Mammalian target of rapamycin (mTOR) and its downstream mediators the eukaryotic translation initiation factor 4E (eIF4E)-binding protein 1 (4E-BP1) and p70S6K were phosphorylated in a time-dependent manner in the presence of PDGF-BB. In the hypophosphorylated state, 4E-BP1 binds to and inhibits the activity of eIF4E. The phosphorylation of 4E-BP1 induces the release of 4E-BP1 from eIF4E, which leads to the subsequent translation of several malignancy-related mRNA that are involved in the growth, survival, and angiogenesis of tumors [31]. In immortalized primary human mammary epithelial cells, mouse eIF4E protein increases translation of human PRDX5 mRNA [32]. Through western blot analysis, we observed that PDGF-B, eIF4E, and p70S6K were significantly increased in the GC tissues, compared with normal gastric tissues. An analysis of the upstream regulators of the PDGF-B pathway using the pathway analysis software helped us to identify more differentially expressed proteins and validate the credibility of our proteomics data.

CALR is a highly conserved endoplasmic reticulum (ER) chaperone protein that participates in various cellular processes. The major functions of CALR inside the ER are protein chaperoning and regulation of Ca^2+^ homeostasis [33]. The correlation between CALR expression levels and tumorigenesis has been extensively studied in various cancers and most related reports have indicated that tumor tissues express significant higher levels of CALR than normal tissues do. In GC, previous studies have reported that positive immunohistochemical staining for CALR was significantly correlated with clinical stages and lymph node metastasis [34]. We also observed that CALR was overexpressed in GC tissues using our label free LC-MS approach. Other ER chaperone proteins were also detected as being differentially expressed between GC and normal gastric tissues in the present research, including PDIA3 and PDIA6. The ER stress pathway is widely considered to be involved in GC development.

S100A6 (calcyclin), a member of the S100 family of EF-hand Ca^2+^ binding proteins, has been implicated in the regulation of cell growth and proliferation. Increased S100A6 level has been observed in many tumors, such as melanoma, colorectal carcinoma, and pancreatic cancer. However, in contrast, S100A6 expression in prostate cancer has been reported to be decreased [35]. Previous research reported that S100A6 could be used as a marker to further subdivide early-stage and late-stage cancer patients into different prognostic groups in GC [36]. However, our study showed a significantly decreased expression of S100A6 in GC tissues. This conflicting data could be due to inherent differences in clinical samples and etiological differences between tumor subtypes among the different studies. The cellular effects of CALR and S100A6 are mediated by the extracellular signal-regulated kinase (ERK) pathways. We found that the expression of total ERK1/2 was not significantly different between GC and normal gastric tissues. However, the phosphorylation and activation status of ERK1/2 were observed significant increase. Therefore, the expression of CALR and S100A6 that we detected in GC tissues may have resulted from crosstalk with other signaling pathways.

CTSD is an intracellular aspartic protease of the pepsin superfamily. CTSD is involved in several physiological functions such as protein degradation, apoptosis and autophagy. Proteomic studies have recently confirmed the upregulation of CTSD in many types of cancer such as nasopharyngeal carcinoma, breast cancer, and colorectal cancer [37, 38]. The overexpression of CTSD has previously been reported in GC using immunohistochemistry assays [39]. Our data also confirmed that CTSD was overexpressed in GC tissues, which is highly concordant with the results of previous studies on GC. Protein kinase B or AKT (PKB/AKT), which is an upstream regulator of CTSD, was also found upregulated in GC tissues in our study.

As illustrated above, we validated 4 differentially expressed proteins (PRDX5, CALR, CTSD, and S100A6) identified in the proteomics analysis, and confirmed PRDX5 and CTSD were regulated by the PDGF-B signaling pathway. PDGF-B and PDGFR-β have been demonstrated to be expressed in many human cancers. Previous reports have indicated that the secretion of PDGF-B by GC cells and the expression of PDGFR-β by tumor-associated stromal cells are associated with angiogenesis, progression, and metastasis [26]. In this study, we observed that both PDGF-B and PDGFR-β were upregulated in GC tissues. In addition, we observed that the knockdown of *PDGF-B* using shRNA inhibited the proliferation of GC cells. The administration of exogenous PDGF-BB recovered the cell proliferation and the reduced expression of protein involved in the PDGF-B signaling pathway in PDGF-B knockdown GC cells. Thus, inhibiting the PDGF-B signaling pathway may be a reasonable approach for the treatment of GC.

The present study has some obvious limitations. Firstly, we identified 87 proteins as being differentially expressed between GC and normal gastric tissues based on the analysis of only 6 patients. Then, we needed to collect multiple specimens for the confirmation of the differential expression of these proteins to avoid heterogeneity. Secondly, other signaling pathways including the mitochondrial dysfunction and ER stress pathways were also involved in GC progression. Therefore, we need to validate more proteins by western blot and immunohistochemistry analysis.

In conclusion, we identified 87 proteins as being differentially expressed between GC and normal gastric tissues by a label-free proteomics approach. GC tissues showed an obvious upregulation of PRDX5, CALR, and CTSD, and a marked downregulation of S100A6. Furthermore, using pathway analysis, we found that upstream regulators including PDGF-B, PDGFR-β, AKT, eIF4E, and P70S6K were obviously increased in the GC tissues compared with the normal gastric tissues. With the experimental validation, we proposed a novel PDGF B regulation network in GC progress. Our findings indicated that the PDGF-B signaling pathway plays an important role in the regulation of GC cell proliferation, and the inhibition of this pathway may be a reasonable approach for the treatment of GC.

## MATERIALS AND METHODS

### Cell culture and tissue samples

The GC cell line SGC-7901 was provided by the Shanghai Institute for Biological Sciences, Chinese Academy of Sciences and was cultured in RPMI 1640 medium supplemented with 10% (v/v) fetal bovine serum and antibiotics at 37°C in a 5% CO_2_ atmosphere. Six pairs of matched primary GC and adjacent normal tissue samples were derived from surgical specimens collected by the same surgeon at the Affiliated Yantai Hospital of Binzhou Medical University. All specimens were quickly rinsed from blood and then frozen immediately in liquid nitrogen and then stored at −80°C until further processing. The 6 cases selected were based on a clear pathological diagnosis of GC and had not received preoperative anticancer treatment. These GC cases comprised 5 men and 1 woman, with an age range of 45-73 years (mean, 59 years). Supplementary Table 1 shows the clinical and pathological data of 6 patients. This study was approved by the Human Research Ethics Committee of Binzhou Medical University (permission number: 2014-013).

### Reagents and antibodies

Goat anti-human PRDX5 polyclonal antibody (AF5724), sheep anti-human S100A6 polyclonal antibody (AF4584), goat anti-human Calreticulin polyclonal antibody (AF3898), goat anti-human Cathepsin D polyclonal antibody (AF1014), mouse anti-human eIF4E monoclonal antibody (MAB3228) and rabbit anti-human P70S6K polyclonal antibody (AF8962) were purchased from R&D Systems (Minneapolis, MN, USA). Rabbit anti-human PDGFR-β monoclonal antibody (ab32570) and Rabbit anti-human PDGF-B monoclonal antibody (ab871409) were from Abcam (Cambridge, UK). Rabbit anti-human Akt polyclonal antibody (sc-8312) and mouse anti-human GAPDH monoclonal antibody (sc-32233) were from Santa Cruz Biotechnology (Dallas, TX, USA). Rabbit anti-human ERK1/2 polyclonal antibody (sc-94) was bought from ZSGB-BIO (Beijing, China). Mouse anti-human β-tublin monoclonal antibody (100109-MM05) was purchased from Sino Biological Inc. (Beijing, China). Bcl-2 antibody (2872), caspase-3 antibody (9662), phospho-Erk1/2 monoclonal antibody (4370), phospho-Akt monoclonal antibody (S4060), phospho-p70S6K antibody (9205) and phospho-eIF4E antibody (9741) were purchased from Cell Signaling Technology (Danvers, MA). PDGF-BB (CYT-501-b) was obtained from ProSpec (Ness-Ziona, Israel). Protein carbonyl contents were assayed using protein carbonyl ELISA kit (ab126287, Abcam).

### LC-MS analysis

All analyses were performed using a QExactive Plus Orbitrap™ mass spectrometer (Thermo Fisher Scientific, Waltham, MA, USA) equipped with a nano-electrospray ion source. Samples were dissolved in water/formic acid (0.1%, v/v), and peptides were separated by reversed phase liquid chromatography using an EASY-nLC™ 1000 system (Thermo Fisher Scientific). A set-up of pre-column and analytical column was used. The pre-column was a 2 cm EASY-column (1D 100 μm, 5 μm C18) (Thermo Fisher Scientific) while the analytical column was a 10 cm EASY-column (ID 75 μm, 3 μm, C18) (Thermo Fisher Scientific). Peptides were eluted with a 90 min linear gradient from 4% to 100% acetonitrile at 250 nL/min. The mass spectrometer was operated in positive ion mode, acquiring a survey mass spectrum with resolving power 70 000 and consecutive high collision dissociation fragmentation spectra of the 10 most abundant ions. The acquired data (.RAW-files) were processed by Maxquant (Version 1.5.0.1) against the Uniprot-Swissprot database using an extracted FASTA file specified for “human” taxonomy. The search parameters included: maximum 10 ppm and 0.02Da error tolerance for the survey scan and MS/MS analysis; enzyme specificity was trypsin; maximum 2 missed cleavage sites allowed; cysteine carbamidomethylation was set as static modification; oxidation (M) was set as variable modifications. The protein identification was based on 95% confidence per protein.

### Pathway analysis

The pathway analysis was performed using Ingenuity^®^ Pathway Analysis (IPA) software (Qiagen, CA). The differentially expressed proteins and their log2-transformed expression ratios were uploaded into the IPA software and the top canonical pathways associated with the uploaded proteins were listed along with the *p* values calculated using a right tailed Fisher’s exact test. The upstream causal network was generated with advance analysis package.

### Real-time polymerase chain reaction (PCR)

For real-time PCR, total RNA was extracted from frozen or RNA later^®^-preserved tissue samples using a high-capacity cDNA reverse transcription kit (TransGen Biotech, Beijing, China), according to the manufacturer’s instructions. Amplification of *GAPDH* cDNA was performed as a control for mRNA content. The following primers were used: *PRDX5* forward: 5′-TCCTGGCTGATCCCACTGG-3′, and reverse: 5′-CAGGGCCTTCACTATGCCAT-3′; and *PDGF-B* forward: 5′-CTCGATCCGCTCCTTTGATGA-3′, and reverse:5′-CGTTGGTGCGGTCTATGAG-3′. Equal amounts of each cDNA were analyzed by real-time PCR with specific primers for *PRDX5* and *PDGF-B* and by EXPRESSSYBR^®^ Green quantitative PCR SuperMix (TransGen Biotech) using a real-time PCR system (Bio-Rad, Hercules, CA, USA). Each sample was measured in triplicate.

### Western blot

The 6 paired of tissue samples and cells were homogenized in the lysis buffer containing protease inhibitors and incubated at 4°C for 30 min. After removing precipitated cell debris by high speed centrifugation (12000 rpm for 10 min), the supernatants were added with loading buffer and then boiled at 95°C for 10 min for denature. Approximately 40 μg of each sample was separated by sodium dodecyl sulfate-polyacrylamide gel electrophoresis on a 10–15% (v/v) polyacrylamide gel. A western blotting system (Bio-RAD) was used to transfer the proteins to polyvinylidene fluoride membrane, which was blocked with 5% (w/v) fat-free milk for 2 h at room temperature and then incubated with an appropriate primary antibody for 12 h at 4°C. The membrane was then washed in Tris-buffered saline containing 0.1% (v/v) Tween^®^ 20 (TBST) 3 times and incubated with an appropriate secondary antibody conjugated with horseradish peroxidase for 2 h. Finally, the membrane was repeatedly washed in TBST and the bound antibodies were visualized using an enhanced chemiluminescence system (Clinx, Shanghai, China). Immunoblot densitometry and normalization was performed using Image-Pro Plus 6.0 software. Normalized intensity values were compared using the non-parametric Mann-Whitney U test (two-tailed, significance threshold P<0.05) in GraphPad Prism software (version 5.01).

### Short hairpin RNA (shRNA) design and transfection

The gene sequence of *PDGF-B* and *PRDX5* was obtained from Genbank (Gene ID: 5155 and 25824). Two sequence-specific shRNAs were designed based on the rules as described elsewhere. The shRNA expressing plasmids were constructed by GenePharma Corporation (Shanghai, China) using pGPU6/GFP/Neo vector. The sequences of the shRNAs targeting *PDGF-B* and *PRDX5* were 5′- GCGGAAGAAGCCAATCTTTAA-3′ and 5′-GCCTGGCACCCAATATCATCT-3′, respectively. An unrelated shRNA sequence with no homology to any human gene was used as a negative control. Transfection was performed using OPTI-MEM^®^ reduced serum media (Life Technologies, Carlsbad, CA, USA) according to the manufacturers’ instructions.

### Cell proliferation assay

The proliferation of SGC-7901 cell line was monitored using an xCELLigence^®^ Real-Time Cell Analyzer Dual Plate (RTCA-DP) system (Acea Biosciences, San Diego, CA, USA). This instrument can measure the proliferation of cells in real-time. Fifty microliters of culture medium was added to each well of an E-Plate 16 (Roche Applied Science, Penzburg, Germany) to obtain equilibrium. Transfected cells were incubated in 6-well plates for 24h and then 1×10^4^ of these transfected cells were seeded in E-Plate 16 in 100μl of culture medium. The E-Plate 16 was then incubated in the RTCA-MP device at 37°C with 5% CO_2_. Measured changes in electrical impedance resulting from cell proliferation on the biocompatible microelectrode coated plate surface were used to calculate as cell proliferation index. The cell proliferation index was calculated automatically every 15min and recorded as a graph of cell proliferation over time.

### Statistics analysis

The protein expression profiling was analyzed with the two-tailed paired Student’s t-test between GC tissue samples and adjacent samples (n=6) with SPSS v20.0 (IBM, Chicago, IL).

## SUPPLEMENTARY MATERIALS FIGURES AND TABLES



## Abbreviations

PRDX5: peroxiredoxin5
CALR: calreticulin
CTSD: cathepsin D
GC: Gastric cancer
CEA: carcinoembryonic antigen
CA 19-9: carbohydrate antigen 19-9
CA72-4: carbohydrate antigen 72-4
MS: Mass Spectrometry
HER2: human epidermal growth factor receptor 2
HNPs 1-3: human neutrophil peptides 1-3
PRDXs: Peroxiredoxins
mTOR: Mammalian target of rapamycin
eIF4E: eukaryotic translation initiation factor 4E
4E-BP1: eukaryotic translation initiation factor 4E (eIF4E)-binding protein 1
ER: endoplasmic reticulum
ERK: extracellular signal-regulated kinase
