# Supplementary material for: Quantitative proteomic analysis of gastric cancer tissue reveals novel proteins in platelet-derived growth factor B signaling pathway
Source: Oncotarget. 2017 Mar 6;8(13):22059–75. doi: 10.18632/oncotarget.15908 (PMC5400646; doi:10.18632/oncotarget.15908)
Supplement: Supplementary file 1 [file oncotarget-08-22059-s001.pdf]

Quantitative proteomic analysis of gastric cancer tissue reveals novel proteins in platelet-derived growth factor B signaling pathway

SUPPLEMENTARY MATERIALS

SUPPLEMENTARY FIGURES AND TABLE

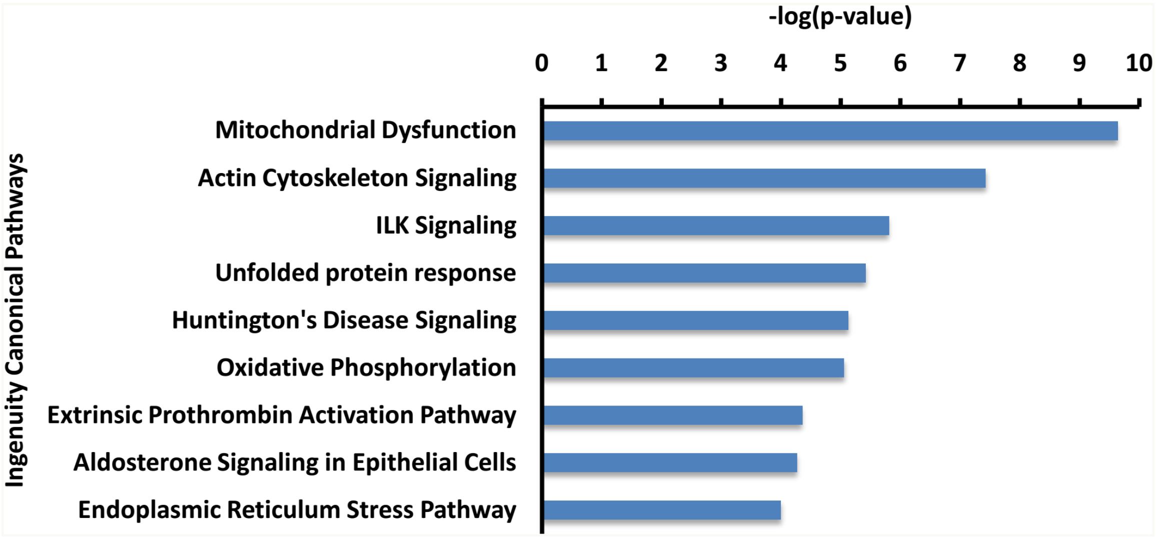

Supplementary Figure 1: The top 10 pathways relevant to the differential expression proteins were shown with the corresponding score (A).

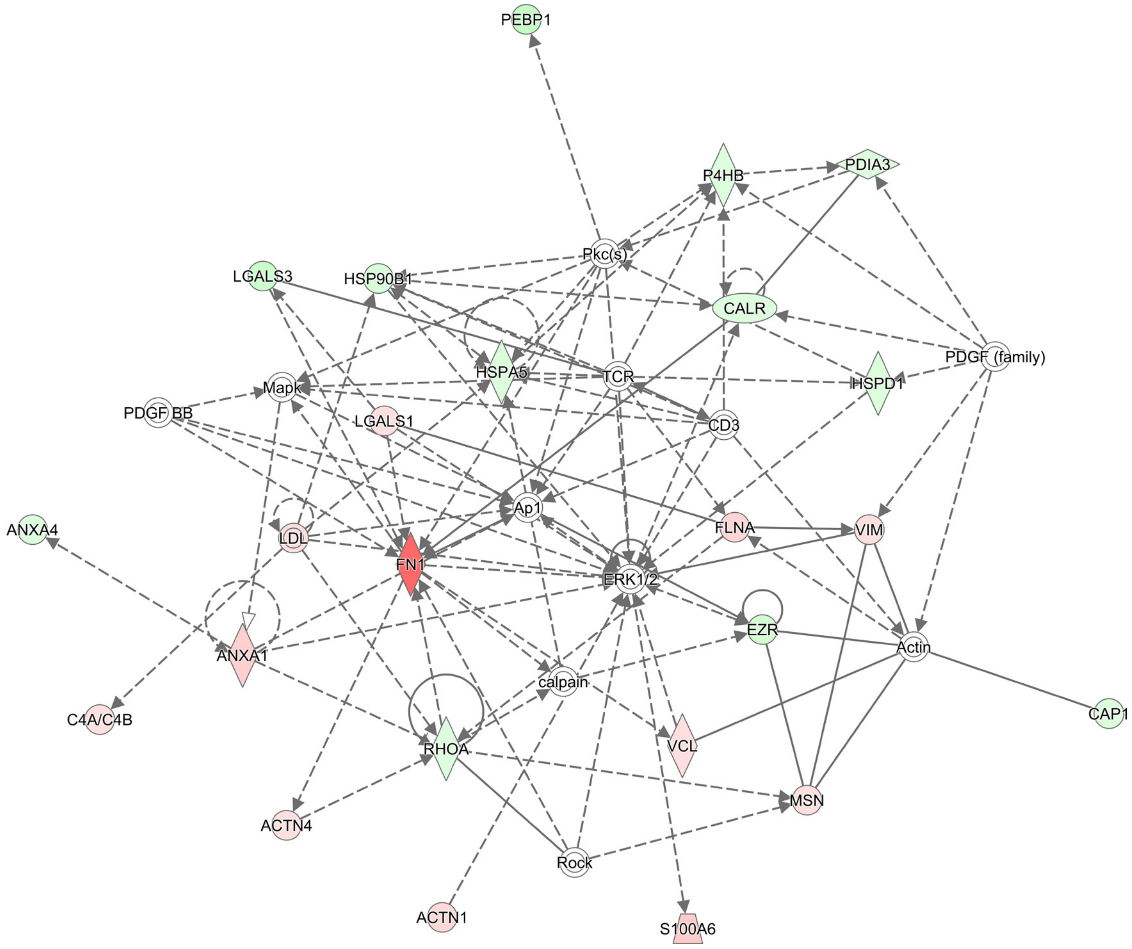

Supplementary Figure 2: PDGF family and PDGF-BB are the two major missing nodes in the network analysis (B).

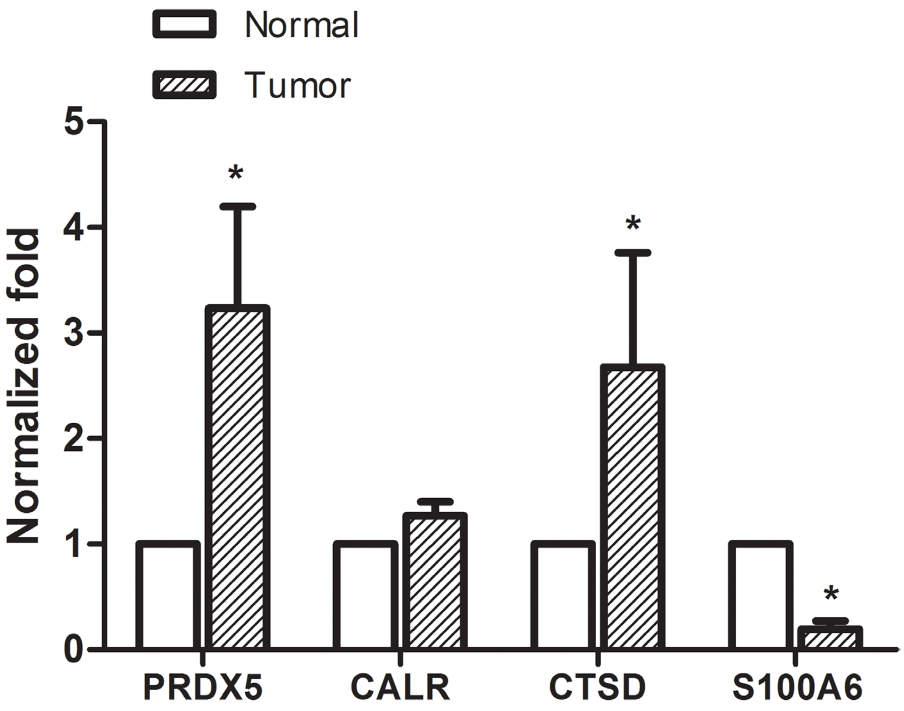

Supplementary Figure 3: Densitometric quantification of blots presented in Figure 3.

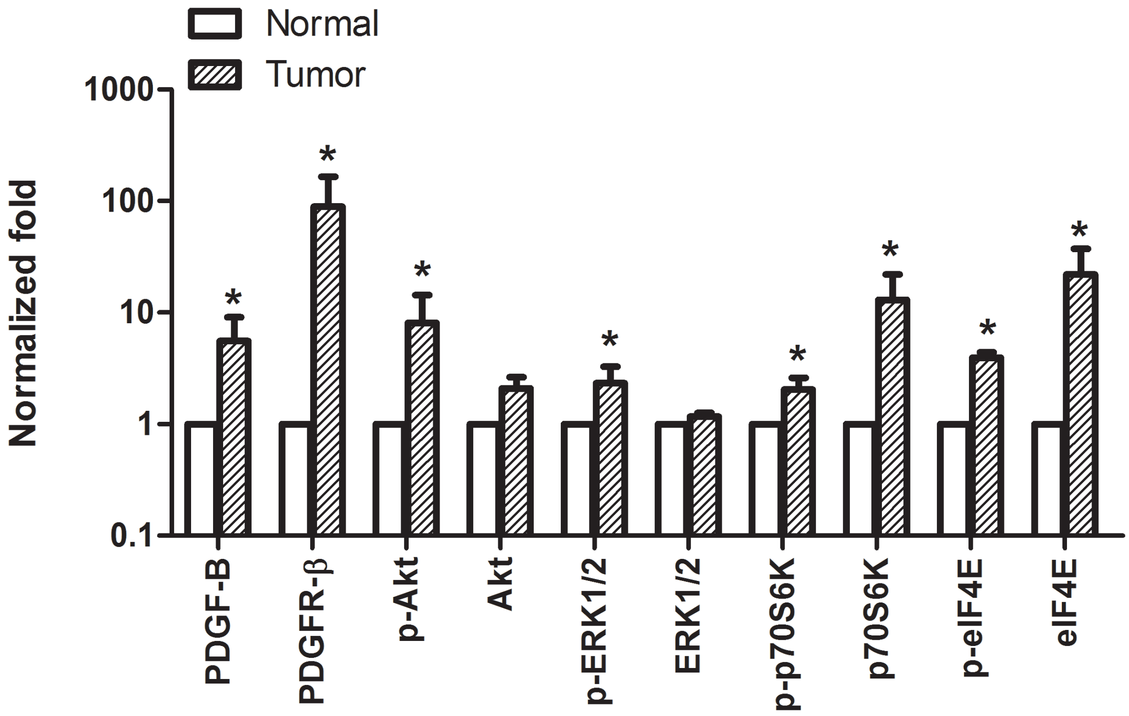

Supplementary Figure 4: Densitometric quantification of blots presented in Figure 4.

Supplementary Table 1: The clinical and pathological data of gastric cancer patients (6 samples)

Sample no.	Gender	Age (years)	Tumor position	pathology	Grade	Stage	TNM	Type
1	Male	61	Gastric body	Adenocarcinoma	3	IIa	T2N1M0	Malignant
2	Male	45	Gastric body	Adenocarcinoma	3	IIb	T3N1M0	Malignant
3	Male	64	Gastric body	Adenocarcinoma	3	Ib	T2N0M0	Malignant
4	Male	54	Gastric body	Adenocarcinoma	3	IIIa	T3N2M0	Malignant
5	Female	57	Gastric body	Adenocarcinoma	3	IIb	T3N1M0	Malignant
6	Male	73	Gastric body	Adenocarcinoma	3	IIb	T2N2M0	Malignant
